# Machine-Learning-Based LOS Detection for 5G Signals with Applications in Airport Environments

**DOI:** 10.3390/s23031470

**Published:** 2023-01-28

**Authors:** Palihawadana A. D. Nirmal Jayawardana, Hadeel Obaid, Taylan Yesilyurt, Bo Tan, Elena Simona Lohan

**Affiliations:** Electrical Engineering Unit, Tampere University, 33720 Tampere, Finland

**Keywords:** 5G signals, Air Traffic Management (ATM), airport areas, Line of Sight (LOS) detection, Non Line of Sight (NLOS), Sounding Reference Signals (SRS), Positioning Reference Signals (PRS)

## Abstract

The operational costs of the advanced Air Traffic Management (ATM) solutions are often prohibitive in low- and medium-sized airports. Therefore, new and complementary solutions are currently under research in order to take advantage of existing infrastructure and offer low-cost alternatives. The 5G signals are particularly attractive in an ATM context due to their promising potential in wireless positioning and sensing via Time-of-Arrival (ToA) and Angle-of-Arrival (AoA) algorithms. However, ToA and AoA methods are known to be highly sensitive to the presence of multipath and Non-Line-of-Sight (NLOS) scenarios. Yet, LOS detection in the context of 5G signals has been poorly addressed in the literature so far, to the best of the Authors’ knowledge. This paper focuses on LOS/NLOS detection methods for 5G signals by using both statistical/model-driven and data-driven/machine learning (ML) approaches and three challenging channel model classes widely used in 5G: namely Tapped Delay Line (TDL), Clustered Delay Line (CDL) and Winner II channel models. We show that, with simulated data, the ML-based detection can reach between 80% and 98% detection accuracy for TDL, CDL and Winner II channel models and that TDL is the most challenging in terms of LOS detection capabilities, as its richness of features is the lowest compared to CDL and Winner II channels. We also validate the findings through in-lab measurements with 5G signals and Yagi and 3D-vector antenna and show that measurement-based detection probabilities can reach 99–100% with a sufficient amount of training data and XGBoost or Random Forest classifiers.

## 1. Introduction and Motivation

In many practical wireless applications involving positioning, communications, or sensing tasks, it is essential to identify the exact position of a User Equipment (UE). Accurate position information can serve multiple purposes, such as location-based beamforming, UE/aircraft tracking to help various dispatcher management operations, and position-based collision detection. The estimation of the position through wireless signals is generally complicated in a real network due to the possible obstructions of the Line of Sight (LOS) path and the predominantly Non Line of Sight (NLOS) propagation as a result of reflections and diffractions from many obstacles encountered throughout the propagation path from the base station to the UE. Therefore, the determination of the existence of a direct LOS path is a fundamental necessity as a starting point in many of the positioning-based studies encountered in the literature.

A recent application area of the Fifth generation of cellular communications (5G)-based positioning and sensing has been in the field of Air Traffic Management (ATM) in small-sized and medium-sized airports [1,2,3,4]. Typical ATM solutions for large-sized airports with heavy traffic rely on Advanced Surface Movement Guidance and Control Systems (A-SMGCS), including Multilateration (MLAT) and Surface Movements Radar (SMR) solutions. However, A-SMGCS solutions have prohibitive costs for smaller-sized airports. At the same time, the air traffic growth also puts the secondary airports under pressure for greater capacity and increased safety. Therefore, low-cost surveillance solutions developed outside the ATM domain and relying on existing infrastructures, such as 5G networks have gained particular attention over the last few years, in particular in EU/SESAR-funded projects such as Emphasis [5], DroC2om [6], or NewSense [2,3]. Nevertheless, there are still very few studies focusing on 5G-based positioning and sensing for ATM applications, as most 5G-based solutions so far have been dedicated to improved wireless communications tasks [5,6]. The research work on 5G applications for positioning, tracking, and/or LOS detection for ATM applications is still in the incipient phase, and there is still a literature gap with respect to suitable LOS detection solutions based on 5G signals; our paper aims to fill in this research gap.

**Figure 1:**
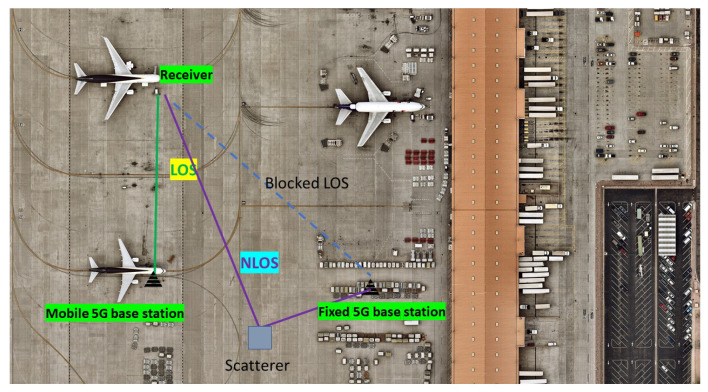
Example of an application area of the studied research question about LOS detection with 5G New Radio (NR). The continuous green line illustrates a LOS signal, the piece-wise continuous magenta line illustrates a NLOS signal, and the dashed blue line illustrates a signal that will be blocked before reaching the receiver.

To emphasize the usefulness and motivation of 5G-based LOS detection in an ATM context, we start with an example of a target scenario where LOS detection algorithms would be beneficial; this is depicted in Figure 1: a small- or medium-sized airport area equipped with fixed or mobile 5G base stations (e.g., some installed in the terminal, some installed on-board the aircraft) and aiming at using 5G signals as complementary solutions to perform ATM-related tasks. Our previous work in [2] explained how 5G-based positioning and sensing could be potentially fused to enhance surveillance in the small-and-medium sized airports; the accuracy of 5G-based positioning and sensing can benefit and be increased if additional information about LOS/NLOS scenarios is available at the receiver. As shown in Figure 1, an LOS scenario means a direct, non-obstructed signal propagation between the transmitter and the receiver. In contrast, an NLOS scenario means an indirect signal propagation due to various obstacles in the signal paths (e.g., scatterers, reflectors, etc.). An example illustrating an NLOS path due to scattering caused by an object in the environment while the LOS path is obstructed by other obstacles is shown in Figure 1.

The main research questions addressed by our paper are: how to perform LOS detection based on 5G Uplink (UL) or Downlink (DL) signals and which feature-extractors and classifier algorithms are the most suitable when dealing with 5G signals. An additional research question is which channel models, from three consecrated 5G channel models in the literature (namely Tapped Delay Line (TDL), Clustered Delay Line (CDL), and WINNER II) are the most challenging in terms of LOS detection.

The novelty of our paper stems from four main aspects: (i) addressing the LOS detection problem in the context of 5G NR, which has been rather poorly addressed so far; (ii) comparing multiple Machine Learning (ML) methods in three realistic channel environments (namely TDL, CDL, and Winner II channel models, under LOS and NLOS conditions); (iii) comparing multiple time- and frequency-domain features and selecting the best feature to characterize 5G LOS/NLOS behaviour; and (iv) validating the findings with in-lab 5G measurements.

The rest of the paper is organized as follows: Section 2 gives a state-of-the-art overview of LOS detection solutions with wireless signals, paying special attention to the prior and scarce work of LOS detection with cellular and 5G signals. Section 3 presents the adopted signal and channel model. Section 4 is dedicated to the methodology adopted in our research, including also a brief overview of the selected ML approaches. Section 5 presents the simulation-based results with three channel types and Section 6 shows the measurement-based results with measurement data collected in our Tampere University laboratory. Last but not least, Section 7 presents the conclusions and the main take-away points of our research.

## 2. State-of-the-Art in LOS Detection

There are several approaches used in scientific research for the detection and separation of the LOS and NLOS propagation paths, which can be mainly categorized into the two classes as mentioned below:

- Model-driven category, where a statistical model followed by thresholding can be used to separate between LOS and NLOS scenarios, e.g., [7,8,9,10,11].
- Data-driven category, where some form of training data for LOS and NLOS cases is available to train some models via feature extraction, followed by some ML classifier stage, e.g., [9,12,13,14,15,16,17,18,19].

The main focus of our paper is on the second above-mentioned category, which, based on literature studies, has been generally found to give more accurate classification results than the first category. Nevertheless, this paper also considers thresholding-based approaches as benchmark cases.

Concerning the model-driven category, the following statistical features and systems have been analyzed in the literature so far: Channel Impulse Response (CIR) kurtosis with the Fourth generation of cellular communications (4G) signals in [7], the Rician factor of the envelope of the received signal in [8,11], or entropy of CIR based on WiFi signals in [10].

With respect to the data-driven category, the following features, ML methods, and systems have been analyzed so far in the literature: Neural Networks (NN) and random forest using uplink reference signal beam-power measurements in 5G and outdoor measurement data in [12] (the focus in there was on positioning accuracy and no LOS detection metric was provided); Convolutional Neural Networks (CNN) applied on raw CIR data of Ultra Wide-Band (UWB) signals in [13] and applied on 5G signals in [19]; various ML methods such as Artificial Neural Networks (ANN), random forest, Support Vector Machines (SVM) were used with Vehicle-to-Vehicle (V2V) signals [14] and random-forest-based classifiers were found to give the best accuracy among the studied methods when trained with extracted temporal features of the received power; SVM used with various temporal features of the UWB received signal, including the kurtosis of its envelope [16]; SVM used with WiFi data and again, various temporal features (kurtosis, skewness, etc.) in [17]; SVM with entropy and kurtosis features and UWB signals in [18], etc.

Both model-driven and data-driven approaches were studied in [9] for WiFi signals and Received Signal Strength (RSS) measurements; threshold-based statistical models were compared with NN models for LOS detection; the NN features included standard deviation of the RSS, as well as kurtosis, skewness, hyper-skewness, and Rician factors. NN showed slightly better LOS detection accuracies than threshold-based algorithms reaching 90% classification accuracy or higher.

A summary of the main classification methods and features used so far for LOS detection in various wireless systems is shown in Table 1. The few methods studied so far in the context of 5G are given in underlined citations [12,19].

The work in [7] focused on CIR-based LOS/NLOS statistical analysis in 4G signals with kurtosis time-domain feature and thresholding, yet no detection probabilities were investigated and the threshold choice was not studied. The authors in [9] addressed the LOS detection problem with WiFi signals and reported approximately an 85–90% LOS classification accuracy. In [10] the LOS, detection problem is addressed with UWB signal measurements. The work in [11] focuses on LOS detection with narrowband signals and Uniform Circular Array antennas and reported detection accuracies range between 73% and 99% with 100 samples/scenario and indoor measurement data. The authors in [12,19] are among the few others who addressed LOS detection with 5G signals and their results are included as comparative benchmarks in Section 6.3. In [13], NLOS, channel classification with CNN is addressed in the context of UWB signals and classification accuracies range between 82.5% and 87.4%. The authors in [17] focus on WiFi signals and reach an LOS detection accuracy of about 95%, while in [18], the focus is on UWB signals. The work in [14] addresses the LOS detection problem with V2V signals and it is also included as a benchmark in our Section 6.3. Table 1 summarizes the state-of-the-art review on LOS detection algorithms.

As seen in Table 1, LOS detection in the context of 5G signals has been poorly addressed in the literature so far; the vast majority of studies concentrated on UWB [10,13,18] and WiFi [9,20] signals. Furthermore, the vast majority of studies applied some form of ML (e.g., neural networks, random forest, support vector machines, etc.) for classification tasks. Our paper aims at solving the yet poorly addressed research issue of the LOS-detection problem in uplink and downlink 5G signals by implementing a comprehensive pool of ML-based approaches (some of them, such as XGBoost, not yet studied in the literature in this context) and by also looking at the thresholding algorithms taken as benchmarks. In addition, various time-domain and frequency-domain features are selected based on 5G correlation functions with Positioning Reference Signals (PRS) and Sounding Reference Signals (SRS) signals in DL and UL configurations, respectively.

## 3. Signal Model

The 5G signal is an Orthogonal Frequency Division Multiplexing (OFDM) signal. We adopt here the most generic model of an Multiple Input Multiple Output (MIMO) system, with $N_{tx}$ antenna elements at the transmitter side and $N_{rx}$ antenna elements at the receiver side; Single Input Multiple Output (SIMO), Multiple Input Single Output (MISO), and Single Input Single Output (SISO) systems are obviously particular cases of MIMO for $N_{tx}=1$ or $N_{rx}=1$ or $N_{tx}=N_{rx}=1$.

The received 5G signal $r_{a_{2}}\left(t\right)$ at the $a_{2}$-th antenna element of the receiver, $a_{2}=1,\ldots ,N_{rx}$ can be modelled according to Equation (1) [21]:

(1) $$r_{a_{2}}\left(t\right)=\sum_{n=0}^{N-1}\sum_{s=0}^{N_{sc}-1}\sum_{a_{1}=1}^{N_{tx}}x_{n,s,a_{1},a_{2}}e^{+j2\pi \frac{n}{T}t}g\left(t-nT_{s}\right)⊗h_{a_{1},a_{2}}\left(t\right)+\eta \left(t\right)$$

where ⊗ is the convolution operator, *N* is the number of considered OFDM symbols, $N_{sc}$ is the number of OFDM sub-carriers, $x_{n,s,a_{1},a_{2}}$ is the *n*-th modulated symbol corresponding to the *s*-th subcarrier, transmitted from $a_{1}$-th transmit antenna, $a_{1}=1,\ldots ,N_{tx}$ and received by the $a_{2}$-th antenna, and belonging to a complex modulation alphabet, e.g., M-Quadrature Amplitude Modulation (QAM) modulation, $T=\frac{1}{\Delta f_{sc}}$ is the symbol interval (before the guard interval) defined according to the sub-carrier spacing $\Delta f_{sc}$, g(·) is the transmitter pulse shape, and $T_{s}=T+T_{GI}$ is the symbol duration after adding the Guard Interval (GI) $T_{GI}$.

In Equation (1), the multipath fading channel is modelled by the CIR $h_{a_{1},a_{2}}\left(t\right)$ corresponding to the wireless channel path between the $a_{1}$-th transmitter antenna element and $a_{2}$-th receiver antenna element. The factor η(t) models the Additive White Gaussian Noise (AWGN) over the channel.

If we assume that the obstacle sizes are much larger than the antenna-element spacing, then an LOS condition can be defined as the situation where all $h_{a_{1},a_{2}}\left(t\right)$ CIRs are in the LOS condition, and an NLOS condition when all $h_{a_{1},a_{2}}\left(t\right)$ CIRs are in NLOS condition. Three different multipath channel models were adopted, as explained later in Section 4.2.

An example of CIR model with *L* channel paths for TDL channels is given in Equation (2):

(2) $$h_{a_{1},a_{2}}\left(t\right)=\sum_{l=1}^{L}\alpha_{l}^{\left(a_{1},a_{2}\right)}\left(t\right)\delta \left(t-\tau_{l}^{\left(a_{1},a_{2}\right)}\left(t\right)\right)$$

where $\alpha_{l}^{\left(a_{1},a_{2}\right)}\left(t\right)$ is the complex coefficient of the *l*-th channel path between antenna elements $a_{1}$ (at transmitter) and $a_{2}$ (at receiver), and $\tau_{l}^{\left(a_{1},a_{2}\right)}\left(t\right)$ is the *l*-th channel path delay between antenna elements $a_{1}$ and $a_{2}$. When there are no phase changes between the same path but different antenna elements, $\tau_{l}^{\left(a_{1},a_{2}\right)}\left(t\right)=\tau_{l}\left(t\right)$.

The Time of Arrival (ToA)-based positioning is typically based on the correlation between a reference signal (here SRS or PRS) with the received signal $r_{a_{2}}\left(t\right)$ [4,22,23]. Examples of correlation outputs under various wireless channel models are provided in Section 4.2. The Angle of Arrival (AoA)-based positioning is typically based on super-resolution approaches such as MUltiple SIgnal Classification (MUSIC), Estimation of Signal Parameters via Rotational Invariance Techniques (ESPRIT), Minimum Variance Distortion-less Response (MVDR), etc. [4,22,23,24].

The presence of NLOS in the wireless channel path deteriorates both the ToA and AoA estimates if the NLOS paths cannot be detected and eliminated from the final positioning solution [24,25]. Recent research in [26] has also proposed ways to harness information from NLOS paths in order to enhance the UE position and orientation information. An accurate detection of LOS/NLOS scenarios would also serve in extracting NLOS specific information that could be used to improve ToA and AoA-based estimation algorithms.

LOS/NLOS detection problem is basically a binary hypothesis testing problem, testing hypothesis $H_{0}$ (LOS is absent or, equivalently, we have an NLOS condition) versus the alternate hypothesis $H_{1}$ (LOS is present). Details on the threshold-based (i.e., model-driven) and ML-based (i.e, data-driven) are further given in Section 4.3 and Section 4.4, respectively.

## 4. Materials and Methods

### 4.1. Overall Methodology

**Figure 2:**
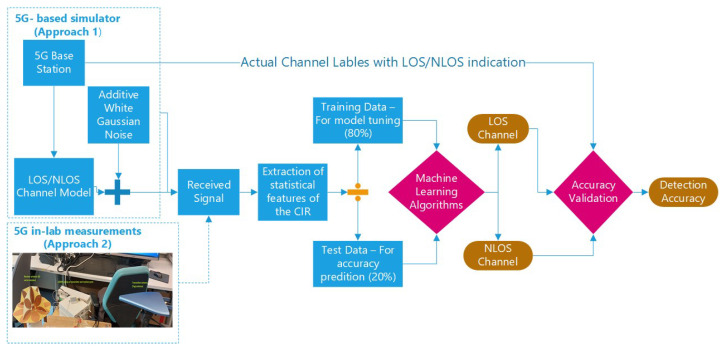
Flowchart of the applied simulation-based and measurement-based methodology.

Figure 2 reflects the overall methodology adopted in our paper: first, a Matlab-based 5G-simulator has been developed, as described in the next sub-section, comprised of a 5G base station, a fading multipath channel model (CDL, TDL, or WINNER II, based on user choice) and AWGN channel. The received signal through multipath fading LOS and NLOS profiles was further analyzed through feature extractors and ML algorithms, as well as via thresholding. A part of the generated data was used for training and the remaining part was used for validation and testing. As a second step, the LOS detection algorithms were also validated with measurement data, as described in Section 6. As seen in Figure 2, first a pre-processing stage based on extracting features is employed; the statistical features are extracted based on the available data and they are fed as inputs to the test and training stages. As shown later, we first investigated five possible statistical features, namely time- and frequency-based correlations, kurtosis, skewness, and Teager-Kaiser energy; then, based on these results, we have selected the best among all tested features and feature combinations and the subsequent results are based on time-based correlation feature only. It is to be noted that, if the configuration of the data fed into the machine learning algorithms is the same (e.g., the same used feature combination, Signal to Noise Ratio (SNR) or SNR ranges, etc.), it is not required to repeat the training process.

### 4.2. Matlab-Based Developed 5G Simulator

**Figure 3:**
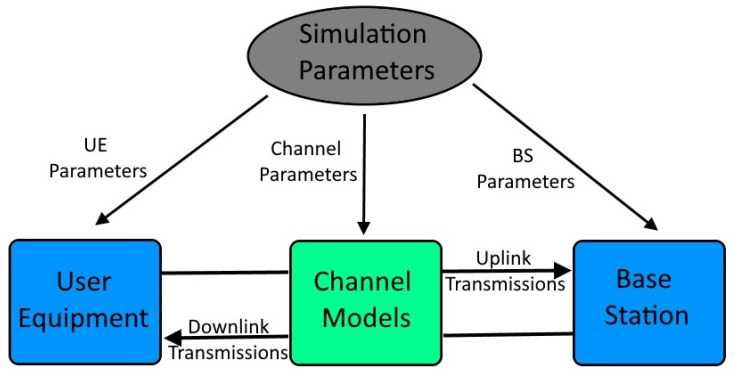
Our 5G simulator software structure.

According to [27], three main types of 5G reference signals can be used for positioning, with one of them for UL positioning and two of them for DL positioning, namely: SRS (UL), Channel Status Information Reference Signal (CSI-RS) (DL), and PRS (DL). Furthermore, according to our team’s previous work in NewSense EU-funded project on 5G-based positioning [3], it has been observed that PRS-based positioning results are better than CSI-RS-based positioning for both ToA and AoA-based positioning. Therefore, without loss of generality in this paper we only focus on PRS 5G signals for DL and on SRS 5G signals for UL. 5G software simulator is designed to simulate secondary airport localization scenarios as realistically as possible by using 5G signal transmissions modelled according to the standards specified in 3GPP releases and realistic channel models. The simulator consists of four main blocks, as shown in Figure 3.

In this realistic simulator, a variety of parameters can be configured. The parameters can be divided into three categories:

- UE Parameters: UE parameters are the simulation parameters related to the transmissions from the UE to the base station, such as UE position in the coordinate system, UE antenna type (Uniform Linear Array (ULA) antennas were used in the simulations and 3D vector antenna (3DVA) antenna was used in the measurements [2,3]), antenna array length (or size), etc. In UL transmission scenarios, UE transmits SRS signals as positioning reference signals. All the configuration parameters of this signal type are also considered as UE parameters in this simulator. These parameters define the allocation of the signals in the resource grid, the modulation of the signals and the UL transmission power. As they do not affect the LOS detection results, they are not described in detail here and they follow 3GPP specifications.
- Base Station (BS) Parameters: BS parameters are the simulation parameters related to the transmissions from the BS and processing of received signals. Some of the BS parameters are the BS position, BS antenna type (ULA antennas were used in the simulations and 3DVA antenna was used in the measurements [2,3]), etc. In a DL transmission scenario, the BS transmits PRS and CSI-RS as positioning reference signals. Our previous studies in [2,3] showed that PRS-based positioning estimators are more accurate than CSI-RS-based positioning estimators, therefore we only focus on PRS reference signals in here for DL scenarios.
- Channel parameters: three multipath fading channel models have been implemented and tested: two of them were based on 3GPP specifications, namely TDL and CDL [28] and one was based on the well-known Winner II channel models [29,30,31]. Some of these parameters are TDL/CDL power-delay profiles, channel delay spread, WINNER II channel propagation scenario, LOS/NLOS flag, etc. Five different TDL channel models, as defined in [28], are used. Two of these models (TDL-D and TDL-E) are used for LOS transmission scenarios and three of these models (TDL-A, TDL-B, and TDL-C) are used for NLOS transmission scenarios. In the simulations, the delay spread parameter is set as 200 ns according to the measurements on small airports in [32]. CDL channels, also described in 3GPP [28] are very similar to TDL channels with the main difference being that, in CDL models, the taps are replaced by clusters. The same five profiles (A-E) as for TDL are also available for CDL channels. Winner II is a channel developed to model a comprehensive range of mobile communication scenarios from short range to wide area [30]. It is possible to simulate spatially defined multiuser MIMO wireless systems. Winner II channel model supports frequencies up to 6 GHz (in our simulations 3.5 GHz was used), bandwidths up to 100 MHz (in the simulations, mostly 9.36 MHz bandwidth was used), 12 indoor and outdoor propagation scenarios, LOS and NLOS propagation, and support for large antenna arrays (we used maximum 4 × 8 antenna arrays).

The Matlab-based simulator was initially built in Matlab R2021a version, starting from the 5G Toolbox and Communications Toolbox in Matlab from MathWorks and WINNER II Channel adds-on. Later on, it was tested also with newer Matlab versions R2022a and R2022b. An in-house simulator was developed to support the three above-mentioned 5G reference signals.

**Figure 4:**
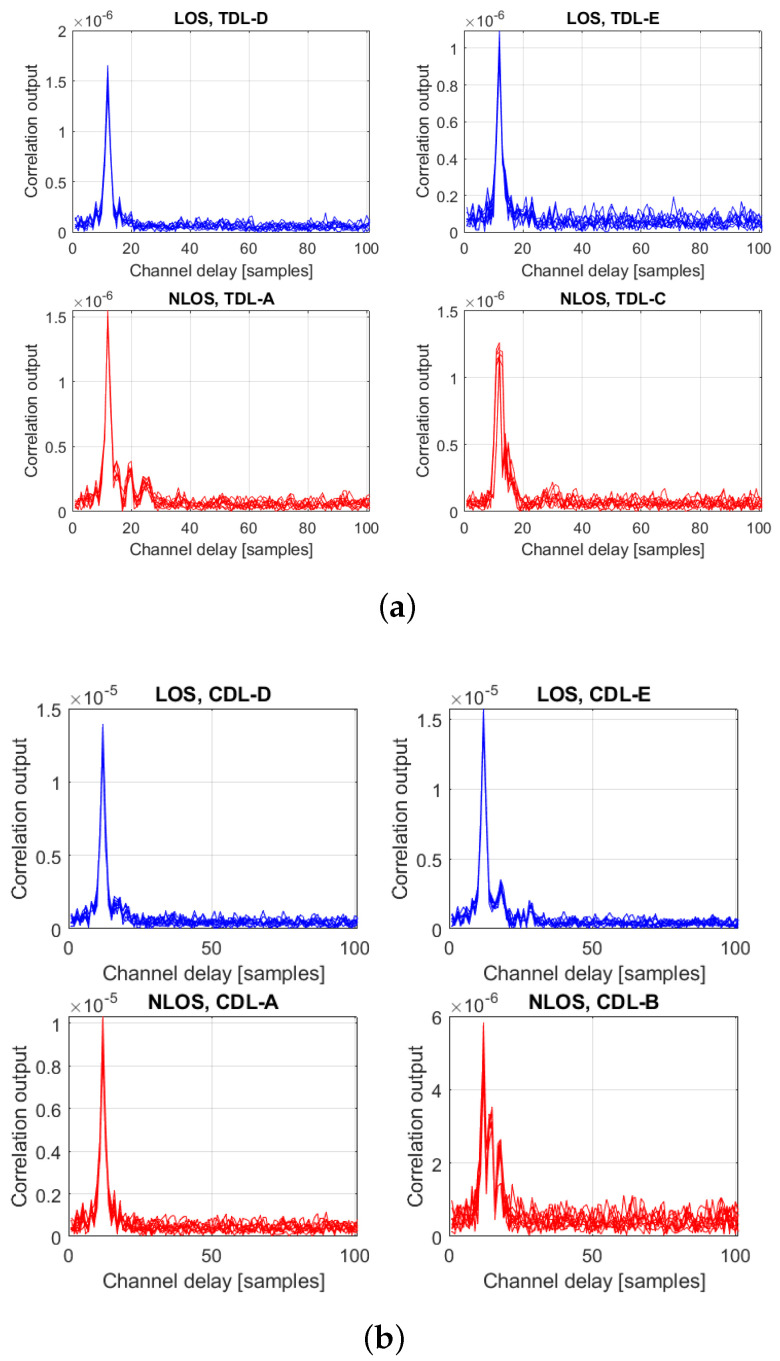
Examples of correlation outputs for PRS signals with various (**a**) TDL and (**b**) CDL channels; 10 random realizations/channel at fixed SNR of 0 dB. Each of the sub-plots corresponds to one of the A to E variants in TDL and CDL. Blue curves are for LOS channels and red curves for NLOS channels.

**Figure 5:**
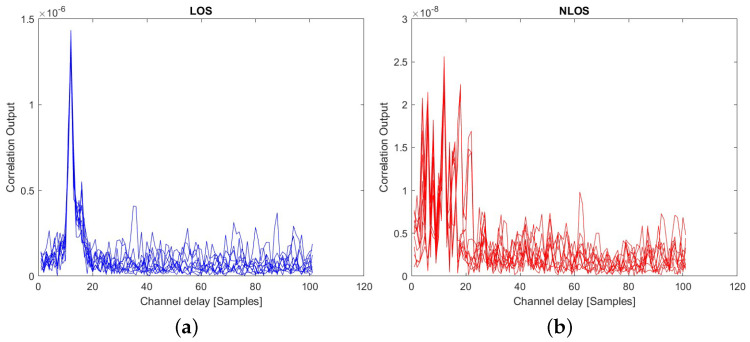
Examples of correlation outputs for PRS signals with WINNER II (**a**) LOS and (**b**) NLOS channels; 10 random realizations/channel at fixed SNR of 0 dB.

The LOS detection relied on the correlation between the incoming signal and the reference positioning signals. Examples of these time-domain correlations are shown in Figure 4 for TDL (left-hand plots) and CDL (right-hand plots) and for two LOS (upper plots) and two NLOS (lower plots) scenarios. Examples based on Winner II channels are shown in Figure 5 for LOS (left-hand plot) and NLOS (right-hand plot) scenarios. In each of these plots, ten different random realizations of signals and channels were considered. Examples based on measurement data are given in Section 6.

### 4.3. Thresholding-Based Benchmark

As previously explained, LOS/NLOS detection is basically a binary hypothesis testing problem. In a model-driven category (i.e., threshold-based), the binary hypothesis testing is based on a decision statistic T, derived from the time or frequency characteristics of the signal and a threshold γ:

(3) $$\begin{array}{c} if\;T\geq \gamma \to choose\;H_{1} \end{array}$$

(4) $$\begin{array}{c} if\;T<\gamma \to choose\;H_{0} \end{array}$$

In our model-driven analysis (used as a benchmark), by analogy with Global Navigation Satellite Systems (GNSS) [33], we built T as the ratio between the maximum time-correlation peak and the next local maximum, and the γ threshold was set to 2.3 for simulation-based data and to 10.5 for measurement-based data. The threshold for the ratio between the maximum peak and the next maximum outside the main lobe for LOS and Ņ channels was selected empirically such as to obtain the best possible detection accuracy for each set of data. By this, it is possible to indicate the highest detection accuracy that could be obtained using the conventional threshold detection algorithms for each scenario. Nevertheless, even under this optimal choice of the threshold, the ML-based approaches gave better performance than the threshold-based approaches.

### 4.4. ML-Based LOS Detection

In a data-driven approach (i.e., based on ML), first the relevant features are selected from the received signal, then the model is trained via various ML algorithms, and then the LOS detection is performed with the trained model.

Based on the literature searches as well as on the results presented in Table 1, three ML-based algorithms have been selected for further analysis with simulation and measurement data and they are described in the following subsections. We would like to mention that additional ML-based tests were done with other NN/ML-based algorithms, but only the three most promising ones were selected for further investigation, due to limited time constraints; the three selected ones are described in the following subsections and they are: XGBoost, Random Forest (RF), and SVM. The choice of RF and SVM has been motivated by the literature findings that we summarized in Table 1, while the choice of XGBoost was motivated by our previous classification work with a different type of data [34], where XGBoost proved to give the most promising results as a classifier.

#### 4.4.1. Extreme Gradient Boosting (XGBoost) Classifier

XGBoost is a gradient boosting algorithm available in Matlab R2022a (Matlab command “fitcensemble”). This algorithm can be used to generate an ensemble of learners for classification [35].

XGBoost (the Matlab version) employs different aggregation methods according to the type and dimensions of the classification problem. Because LOS detection is a binary-classification problem with two classes, the “AdaBoostM1” aggregation method, which is optimized for such classification, was used in here.

XGBoost method has previously proven very good results with other data types used in our research, such as loneliness data [34], but it has not been used so far, to the best of the Authors’ knowledge, in the context of LOS detection with 5G signals. In the context of 5G, the XGBoost algorithm has been used so far mainly for security purposes, such as for detecting the Distributed Denial of Service attacks [36] or for antenna optimization [37].

#### 4.4.2. Random Forest Classifier

The RF classifier was implemented via Matlab “fitensemble” used in conjunction with the bagging aggregation method “bag”.

As the first step in the bootstrap aggregation (bagging) ensemble-learning method, it is required to generate many bootstrap replicas of the original data set, which would contain a random selection of the predictors within each replica. These bootstrap replicas will operate on a randomly selected number of observations. Some of the selected observations will have multiple copies of those included in a single bootstrap replica. The decision trees will be grown on these replicas, with the classification decision of each tree being made only by a randomly selected number of predictors. After the classification is completed by each tree, the final classification decision will be obtained by a majority vote in favour of one of the classes.

The RF method has been previously used for LOS detection in [12,14]. In [12], the LOS, detection is briefly addressed with 5G signals and it will be the basis of our benchmark comparisons in Section 6.3. The work in [14] focuses on LOS detection in V2V communications and no specifics from 5G positioning reference signals are used.

#### 4.4.3. Support Vector Machines (SVM)

The objective of SVM-type classifiers is to find the optimum hyperplanes between the subjective classes as the decision boundaries, in order to classify the different observation samples accurately. Thus, the SVM algorithm is mostly suitable for binary classifications and it would select the hyperplane that maximizes the margin between the two classes. The detection of LOS/NLOS condition of 5G wireless channels falls into the standard category of binary classification for which the SVM algorithms are defined, and thus they are an obvious selection. The fitcsvm algorithm was chosen by us in Matlab for binary classifications. In SVM classifications, the scenarios which involve multiple classes are reduced into a sequence of binary classification problems. In scenarios where the classification cannot be solved by linear segregation of the predictors of the observation data, these features could be transformed by utilizing a different kernel function such as the Gaussian kernel (fine, medium, coarse) or the polynomial kernels (quadratic, cubic). These kernel functions would map the data samples into a higher dimensional vector space which could make it easier to segregate and categorize the data. By selecting a suitable kernel function, it is possible to handle also nonlinear classification problems using SVM. The selection of the suitable kernel function, and the kernel scale depends on the number of predictors and on the characteristics of the observation data samples such as the amount and the dissimilarity; in our case, we chose the best kernel in an empirical manner, testing with the available data. Table 2 specifies the kernel scale of different Gaussian SVM kernel functions with respect to the number of predictors *p* as well as the subtle differences between the three kernel functions that we used in our data analysis.’

**Figure 6:**
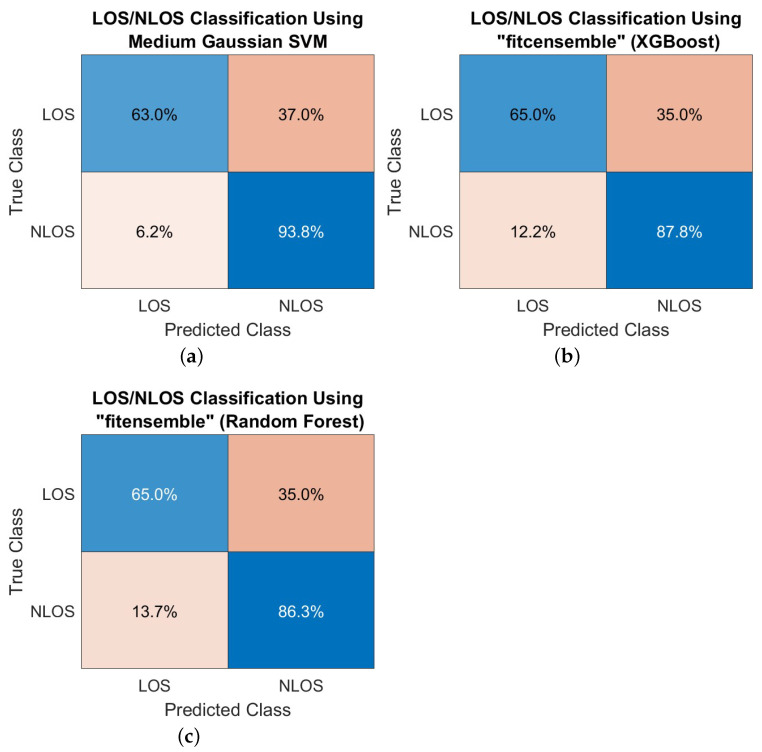
Examples of confusion matrices for (**a**) medium Gaussian SVM, (**b**) XGBoost and (**c**) Random Forest methods.

An example of how the detection probabilities are computed is illustrated in Figure 6. The final detection probability is taken as the average over the diagonal values of the confusion matrices, which show which is the probability to classify a class (LOS or NLOS) into its own (correct) category (diagonal values) or in the opposite category (anti-diagonal values). The examples shown in Figure 6 were obtained with a DL 5G configuration, using PRS reference signals and TDL wireless channels.

## 5. Simulation-Based Results

### 5.1. Simulation Parameters

The Matlab-based simulator was run with the main parameters listed in Table 3. We recall that $N_{RB}$ is number of the resource blocks, the $N_{scprb}$ is the number of sub-carriers per resource block, and $\Delta f_{sc}$ is the sub-carrier spacing.

First, the impact of the choice of various features was studied with TDL and CDL channels and PRS signals and the results are shown in Section 5.2. Due to the significant higher amount of time to generate Winner II channels and to test them with various features, and based on the fact that the same conclusions were drawn from TDL and CDL channels, the Winner II channels were not considered in Section 5.2, but only in Section 5.3.

### 5.2. Feature Selection

The first step in our analysis has been to test which feature or combinations of features give the most promising LOS detection results. For this scope, five features have been selected, based on prior literature studies as shown in Table 1:

- Time correlation—this is the most straightforward feature and has been illustrated, for example in Figure 4;
- Fourier transform of the time correlation, as a representative of frequency-domain characteristics;
- Kurtosis;
- Skewness: typically, skewness is higher for NLOS channels than for LOS channels;
- Teager-Kaiser transform (TK)—this transform was selected based on our previous work on feature identification in a GNSS context [46].

For a fair comparison, only the SVM algorithm has been used in this first stage. Furthermore, since the Winner II simulator is very slow, we only focused on TDL and CDL models. The results in both TDL and CDL channels consistently pointed out the same conclusion that a single feature based on time correlation gives the best results. The results are shown in Table 4. The best results, according to the number of features are emphasized in bold-faced letters and they correspond to a single feature, namely time-based correlations. Few other combinations, also including time-based correlations are also achieving the maximum accuracy, but combining many features has proved to be sub-optimal in our case, most likely due to several effects: (i) some features, such as kurtosis and skewness, give very poor results when being used alone, so it is expected that they also deteriorate (even if slightly) the results when used in combination with other features; (ii) many of these features have string correlations between them. Thus, putting them together, it is intuitively not likely to enhance the results much (if at all). From now on, we will focus only on single-feature approaches, using the time-based correlations of 100 samples window size as the features to be input to ML classifiers.

### 5.3. ML Algorithm Selection

After the initial small-scale tests when some of the ML algorithms were discarded as not having enough performance, we have selected three ML-based algorithms for detailed investigation, namely XGBoost, Random Forest and an SVM implementation.

The results are shown in Table 5. Both XGBoost and SVM classifiers work very well with various channel profiles and various reference signals; the random-forest-based estimator is slightly worse than XGBoost and SVM ones. The best LOS classification results are achieved with the most complex channel model, namely the Winner II channel model, as this complexity basically means that Winner II is the channel model richest in features among the considered ones. The performance in TDL and CDL channels is also according to the richness of features (or complexity); better results are achieved for the more complex model among the two, namely for CDL model. Furthermore, as seen in Table 4, the data-driven ML-based LOS detection outperforms the model-driven threshold-based detection with up to 28%.

### 5.4. SNR Impact

The SNR impact has been tested next and the results for CDL and TDL channels and PRS signals are shown for reference in Table 6. The average values from the last column in Table 6 were not computed by averaging the data in the other columns, but by generating 5G data at a variable SNR, both in the training and test data and computing the LOS detection accuracy under this variable/unknown SNR scenario. As the mean over the individual SNRs is close to the LOS detection accuracy when variable SNR is used in the training and test data, from now on we will show only the results with variable SNR when simulation-based data is used. These results, as seen in Table 6 are indeed also representatives for fixed SNR scenarios, yet a variable SNR assumption is more realistic, as the wireless channels are seldom operating at constant SNR.

### 5.5. Sample Size Impact

Since the number of training samples has a direct effect on complexity, one question is how to choose a good number of training samples. Table 7 shows the impact of increasing the overall number of training samples for TDL channels with PRS signals using the time-based correlation feature. Here, we used 5 5G base stations, with three of them under NLOS conditions (with various NLOS channel models) and two of them under LOS conditions, again under two LOS channel models, as there are five types of TDL channel modes, namely TDL-A to TDL-E. This means that *n* samples from Table 7 are equivalent with n/5 samples per base station or channel model; e.g., a training dataset of 2500 samples meant that we generated 500 samples per each of the five TDL channel models (TDL-A to TDL-E).

From Table 7, we see that with 1000 samples/channel (i.e., *n* = 5000, the fluctuations in the LOS detection accuracy are already less than 1%; with an increased number of training samples, both the complexity and the simulation times are increasing. For a good tradeoff between the samples size and simulation times, we chose in what follows 1000 samples/channel; also, in order to have a balanced number of LOS and NLOS channels, we only used four out of the five available TDL and CDL channels, with two of them under LOS condition and the other two under NLOS condition. This gave us n=4000 for CDL and TDL channels. For Winner II channels, as there was only one LOS and NLOS channel model, we used a total of n=1600 samples, meaning 800 samples/channel; this slightly smaller number was chosen for the sake of reasonable simulation times, as the simulations with Winner II channels are significantly slower than those under CDL and TDL channels. The choice of the sample size is many times application and channel-dependent, not only algorithm dependent; for example, the same ML algorithm may show different sensitivity for different data. Additional discussions on how to set the size of the training data set can be found, for example, in [47].

## 6. Measurement-Based Results

### 6.1. Measurement Setup

**Figure 7:**
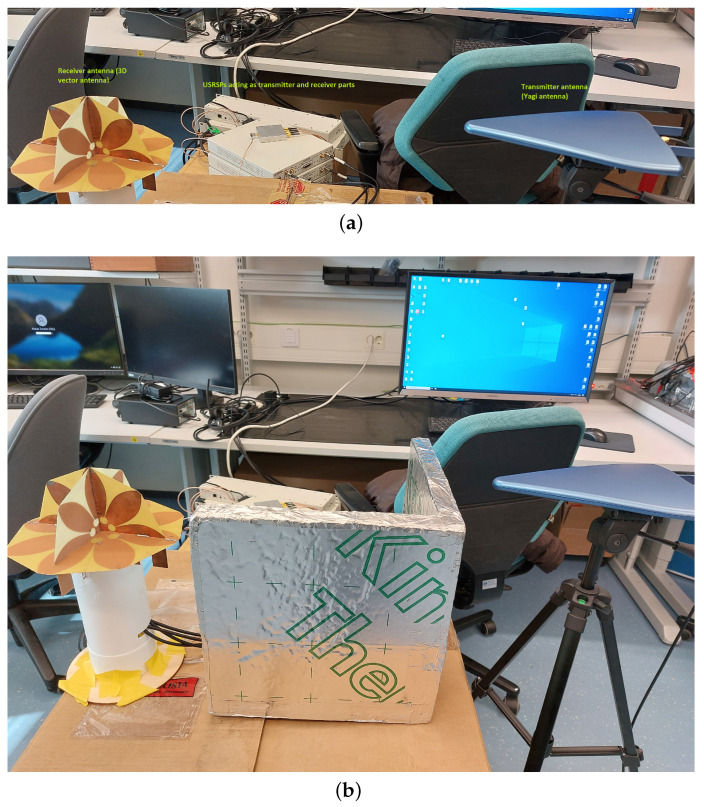
(**a**) LOS and (**b**) NLOS in-lab setup with 5G SRS signals, Yagi antenna at the transmitter and 3DVA at the receiver.

The in-lab measurements for LOS and NLOS were done using National Instruments Universal Software Radio Peripheral (USRP), as illustrated in the setup in Figure 7. Three USRPs (two NI USRP-2954R and one NI USRP-2953R) were set in a synchronized setup using an OctoClock CDA-2990 from National Instruments. One USRP was used as a transmitter (TX) and the other two were used as receiver channels (RXs); two USRPs were needed as the receiver antenna had 4 ports and each USRP had 2 ports only. One PC was connected to all three USRPs, using one CPS-8910 and one PCIe adapter. LabVIEW 2021 was used as the software which provided the graphical user interface to and from the USRPs. An 3DVA with eight ports was used as a receiving antenna, among which four ports were connected to the two receiver USRPs (and four ports remained unused, as the four channels were enough for our studies). The justification for using an 3DVA can be found in our previous work in [2]. A Yagi antenna with 5 dBi antenna gain and a frequency range between 700 MHz and 6 GHz was used as a transmitter. The measurements were done at 2.1 GHz carrier frequency. Five different SRS 5G signals with different configuration parameters were used for LOS and NLOS scenarios. For each SRS 5G signal, the I/Q data was collected from the four-ports of the 3DVA in both LOS and NLOS scenarios.

**Figure 8:**
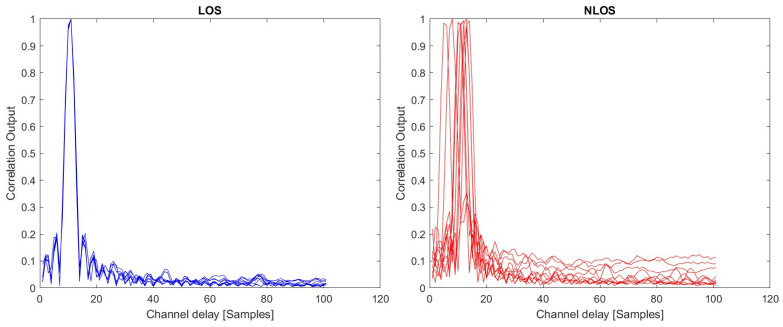
Examples of correlation outputs for SRS signals with measurement data; (**left**): LOS scenarios; (**right**): NLOS scenarios.

Examples of correlation outputs for ten measurements in LOS and ten measurements in NLOS scenarios with SRS 5G signals are shown in Figure 8.

### 6.2. LOS Detection Results with Measurement Data

The LOS detection results with measurement data are shown in Table 8. In total, 100 measurements per LOS/NLOS scenarios were conducted and they have been split into x% training data and (100−x)% test data, with *x* values between 80% and 10%, as shown in Table 8. As expected, the more training data we have, the better the results. Nevertheless, the results were consistently good even with 10% training data for XGBoost and SVM algorithms. The Random Forest algorithm proved highly sensitive to the amount of available training data (i.e., highly deteriorating in performance when the amount of training data decreased). Therefore, RF is not a promising algorithm in the context of LOS detection unless a large amount of training data is available.

### 6.3. Benchmark Results from Literature

As already mentioned in our introduction and state-of-the-art review, to the best of our knowledge there are still very few published studies about LOS detection with 5G signals. This section compares our findings with the limited work reported in the literature so far. Nevertheless, LOS detection has been studied in the literature so far with various other wireless signals, such as UWB or WiFi. Table 9 shows five selected comparative results reported in the literature so far, with two of them also studied in a 5G context. As seen in Table 9, our obtained results are similar to or slightly better than those reported in the literature so far. It is to be noticed that all the comparative results we found on LOS detection are based on measurement data; we could not find any comparative benchmarks for LOS detection relying on simulated data with TDL, CDL or Winner II channels, therefore our paper gives a more comprehensive view of LOS detection algorithms than what exists in the literature so far, by addressing both simulation-based and measurement-based experiments.

### 6.4. Complexity Considerations

In terms of complexity, clearly, the threshold-based approaches are less complex than ML approaches as they do not require complex operations and heavy training databases, yet they do not reach the performance needed for LOS detection in applications such as ATM. Among the considered ML approaches, it is generally understood that the XGboost approach has the lowest complexity, followed by RF, and the SVM is the most complex among the three. In terms of stability, the XGboost is also known to be more stable than SVM [48]. Complexity numbers are shown in Table 10 [49], where *n* is the number of training samples (e.g., in our case n=4000 for TDL and CDL and n=1600 for Winner II simulations), *p* is the number of features (e.g., in our case 101 for time correlation features), $n_{t}$ is the number of trees, which is an intrinsic parameter of the model, and $n_{sv}$ is the number of support vectors, which is also an intrinsic parameter of the model.

Based on our results, XGboost has shown the best tradeoff between accuracy and complexity and it is the recommended one, as discussed in the next section.

## 7. Conclusions

This paper analyzed and compared ML-based LOS/NLOS detection mechanisms with 5G signals and scenarios. First, the most suitable feature-extraction transform to be used with ML was found to be based on the time correlations between the 5G received signal and the reference positioning codes, namely PRS in DL configurations and SRS in UL configurations. Secondly, among the investigated ML approaches, the XGBoost and the SVM with medium Gaussian kernels gave the best results with both simulated and measurement-based data. By taking into account also the complexity and stability of the algorithms, we recommend XGBoost with time-domain features as the main LOS detection algorithms with 5G signals. The simulations relied on three well-known channel models for 5G signals: TDL, TDL, and Winner II channels. The TDL is the ’simplest’ one, in the sense that it has a lower amount of features compared to CDL and Winner II, in terms of antenna-array characteristics, of the channel propagation, and of the clustering profiles. In order to obtain a good diversity of the results, we used ULA antenna array models in the simulations and a 3DVA antenna for measurements and both gave consistent results. Due to their simplicity, it was shown that TDL channels are the most challenging in terms of LOS detection, with detection accuracy slightly worse than for CDL and Winner II channels.

The detection accuracies based on ML went as high as 98% for simulation-based data and 100% for measurement data. The studied ML approaches showed significantly better performance (up to 28% better) than the threshold-based approaches. The better accuracy in the measurement-based data than in the simulated-based data is explained by the higher SNR in the measurement data (i.e., SNR greater than 10 dB, due to measurements and calibration conditions), while the simulation-based results were given as the average values over a variable SNR, ranging between −20 dB and 0 dB.

The in-lab measurement datawas also made available in open access at [50,51]. Further research will focus on integrating the LOS detection blocks into the 5G-based airport surveillance solutions in order to improve their positioning and sensing performance.

## Figures and Tables

**Table 1:** Feature-algorithm combinations used in the literature for LOS detection in various wireless systems; those also analyzed in a 5G context are shown underlined. N/A = not available.

Feature vs. Algorithm	Raw Data	Kurtosis	Skewness/	Rician Factor	Entropy
	(CIR, Power, …)		Hyper-Skewness		
Thresholding	[9]	[7]	[20]	[11]	[10]
ML: NN/ANN/CNN	[13], [19]	[9,14]	[9,14]	[9,14]	N/A
ML: random forest	[12]	[14]	[14]	[14]	N/A
ML: SVM and variants	N/A	[14,17,18]	[14,17]	[14,17]	[18]

**Table 2:** Comparison of three SVM Gaussian kernels used in our studies.

Kernel Function	Recommended Kernel Scale	Model Description
Fine Gaussian SVM	$\sqrt{p/4}$	Very detailed distinctions between the classes. Could cause overfitting of the model if used erroneously
Medium Gaussian SVM	$\sqrt{p}$	Medium level distinctions between the classes.
Coarse Gaussian SVM	$4\sqrt{p}$	Coarse distinctions between the classes. Could cause underfitting of the model if used erroneously.

**Table 3:** Simulation parameters.

Parameter	Value	Justification
Carrier frequency $f_{c}$	3.5 GHz	Typical cmWave 5G frequency band [38,39,40,41,42]
Sampling rate	130 MHz	Selected high enough to allow the extraction of relevant features, yet low enough to allow feasible simulation times
Sub-carrier spacing $\Delta f_{sc}$	15 kHz	According to 5G specs [27]
Number of resource blocks $N_{RB}$	52	According to 5G specs [27] for SRS and PRS
Number of sub-carrier per resource blocks $N_{scprb}$	12	According to 5G specs [27]
Bandwidth *B_W_*	9.36 MHz	Computed from *B_W_* = *N_RB_* ∗ *N_scprb_* ∗ Δ*f_sc_*
Base station ULA array length	8	Typical base-station array length value [12,14,43]
UE ULA array length	4	Typical UE array length value [19]
Maximum tx-rx distance	3500 m	Based on small-sized Muret airport area studied by us previously in [2,3]
Reference signal	Downlink PRS or uplink SRS	The third possible positioning reference signal, downlink CSI-RS has been discarded as giving worse result than PRS, based on our previous studies [2,3]
Number of subcarriers	8192	This is also the FFT length at the receiver; it must be high enough for large $f_{s}$; similar values can be found, for example in [44]
Number of processed frames per random iteration (each frame has 10 ms)	1	Chosen for moderate simulation times; can be increased without loss of generality
Time- correlation window length	100 samples	Chosen for moderate simulation times; can be increases without loss of generality
Number of Monte Carlo runs per LOS or NLOS scenario	between 500 and 50,000; value 1000 was used for most of the tests (if not specified otherwise), after studying the impact of the number of iterations	Chosen empirically, as explained here
TDL channel types	TDL-A (NLOS), TDL-B (NLOS), TDL-C (NLOS), TDL-D (LOS), and TDL-E (LOS)	TDL has been used before for 5G studies [39]
CDL channel types	CDL-A (NLOS), CDL-B (NLOS), CDL-C (NLOS), CDL-D (LOS), and CDL-E (LOS)	CDL has been used before for 5G studies [45]
Winner II channel types	11 C2 (Urban macro-cell) LOS and NLOS	Winner II has been used before for 5G studies [41]
SNR range	Uniform distribution between −20 dB and 0 dB	Typical nominal values for 5G SNR [43]

**Table 4:** Impact of feature selection on the detection accuracy. PRS signals, average results for SNR uniformly distributed between −20 dB and 0 dB. Highest values are emphasized in bold-faced letters.

Features	TDL Channel	CDL Channel
1 feature: time-based correlation	**82%**	**89 %**
1 feature: FFT of time-based correlation	76%	83 %
1 feature: kurtosis	69%	69 %
1 feature: skewness	67%	67 %
1 feature: TK	78%	79%
2 combined features: time correl + FFT	80 %	87 %
2 combined features: time correl + kurtosis	**82%**	87%
2 combined features: time correl + skewness	77%	83 %
2 combined features: time correl + TK	**82%**	87%
2 combined features: FFT +kurtosis	78 %	83 %
2 combined features: FFT + skewness	77 %	83 %
2 combined features: FFT + TK	78 %	84 %
2 combined features: kurtosis + skewness	78 %	78%
2 combined features: kurtosis + TK	79 %	79 %
2 combined features: skewness + TK	79%	79 %
3 combined features: time + FFT + kurtosis	80%	87 %
3 combined features: time + FFT + skewness	80%	87%
3 combined features: time + FFT + TK	81%	87%
3 combined features: time + kurtosis + skewness	**82%**	**89 %**
3 combined features: time + kurtosis + TK	**82%**	87%
3 combined features: time + skewness + TK	**82%**	87%
3 combined features: FFT + skewness + kurtosis	78%	83%
3 combined features: FFT + kurtosis +TK	79%	84%
3 combined features: FFT + skewness +TK	79%	84%
3 combined features: kurtosis + skewness +TK	79%	79%
4 combined features: time + FFT + kurtosis+ skewness	81%	87%
4 combined features: time + FFT + kurtosis+ TK	81%	87%
4 combined features: time + kurtosis+ skewness+ TK	**82%**	87%
4 combined features: FFT + kurtosis+ skewness+ TK	79 %	84%
All 5 combined features: time + FFT + kurtosis + skewness + TK	81%	87%

**Table 5:** LOS detection accuracy ([%]) with simulated data, using three ML approaches and a benchmark threshold-based approach. Bold-faced numbers show the best results per channel type.

Algorithm & Channel Type	TDL Channel and PRS Signal	TDL Channel and SRS Signal	CDL Channel and PRS Signal	CDL Channel and SRS Signal	Winner II Channel and PRS Signal	Winner II Channel and SRS Signal
XGBoost	80%	78%	88%	**86%**	**98%**	**97%**
Random Forest	79%	78%	87%	85%	**98%**	96 %
Medium Gaussian SVM	**82%**	**80%**	**89%**	**86%**	**98 %**	96 %
Threshold-based	67%	64%	66%	61%	75%	72%

**Table 6:** Example of the SNR impact on the LOS detection accuracy ([%]) with simulated data.

Channel Model and Signal Type	ML Algorithm	SNR = 0 dB	SNR = −0 dB	SNR = −10 dB	SNR = −15 dB	SNR = −20 dB	Variable SNR from 0 dB to −25 dB
CDL, PRS	Medium Gaussian SVM	100%	100%	96%	83%	61%	90%
CDL, PRS	XGBoost	100%	99%	97%	82%	58%	89%
CDL, PRS	RF	100%	100%	96%	82%	60%	87%
TDL, PRS	Medium Gaussian SVM	100%	99%	89%	60%	52%	82%
TDL, PRS	XGBoost	100%	99%	89%	58%	54%	80%
TDL, PRS	RF	100%	99%	88%	60%	56%	79%

**Table 7:** Example of the impact of the number of training samples on the LOS detection accuracy ([%]) with simulated data; TDL channels; PRS signals; variable SNR between −20 dB and 0 dB.

ML Algorithm	*n* = 500	*n* = 2500	*n* = 5000	*n* = 10,000	*n* = 25,000	*n* = 50,000
Medium Gaussian SVM	78%	80%	82%	82%	83%	83%
XGBoost	78%	80%	80%	79%	79%	79%
RF	75%	76%	79%	79%	79%	79%

**Table 8:** LOS detection accuracy ([%]) with measurement data and ML approaches; threshold basedapproaches are taken as a benchmark.

Algorithm	80% Training + Validation—20% Testing	50% Training + Validation—50% Testing	20% Training + Validation—80% Testing	10% Training + Validation—90% Testing
XGBoost	99%	**99%**	**97%**	**94%**
Random Forest	**100%**	95%	65%	55%
Medium Gaussian SVM	96%	92%	92%	91%
Threshold-based	72%	72%	72%	72%

**Table 9:** Comparison with other results from the literature about LOS detection with 5G signals.

Reference	Reported Results	Comparative Notes
[12]	Up to 88% NLOS detection probability with 5G measurement data at 15 GHz carrier frequency	The work in [12] focuses on positioning, not on LOS detection and the measurement conditions are different from ours, thus a direct comparison is hard to make. With our measurement-based results, we got a minimum of 94% detection probability with XGboost, even with only 10% of training data
[14]	Between 91% and 99% LOS detection accuracy with V2V measurement data at 5.9 GHz carrier frequency and about 50–50% split between training and testing data	The measurements in [14] included several scenarios and the achieved LOS detection accuracy varied according to the scenario; their maximum achieved LOS detection accuracy of 99% is comparable with our best achieved accuracy with 50–50% data split, as seen in Table 8.
[19]	Between 91.5% and 93.3% LOS detection accuracy with indoor 5G measurements at 3.5 GHz	These measurements are the closest to our measurements in terms of carrier frequency and indoor scenario, yet the percentages of training and testing data are not given in [19] for a direct comparison. Our measurement-based maximum detection probabilities ranged between 94% and 100% for training data between 10% and 80% and thus are outperforming the values reported in [19].
[9]	Between 85% and 90% LOS detection accuracy with measured WiFi signals	The results in [9] are not exactly comparable with our work as they use different types of signals (WiFi and not 5G) but their reported maximum accuracies are below what we obtained with our measurement data and close to what we obtained with our simulation-based data.
[11]	between 82.5% and 87.4% LOS detection accuracy with measurements performed with UWB signals and SVM and CNN classifiers	Our SVM-based accuracies reach 82%, 89%, 96% and 98% with TDL, CDL, Winner II channels and 5G measurements, respectively, so they are very promising also when compared with UWB results.

**Table 10:** Complexity of the considered ML approaches.

Algorithms	Training	Prediction/Testing
XGBoost (typically lowest complexity)	$O(npn_{t})$	$O(pn_{t})$
RF	$O(n^{2}pn_{t})$	$O(pn_{t})$
SVM (typically highest complexity)	$O(n^{2}p+n^{3})$	$O(pn_{sv})$

## Data Availability

Our in-lab measurement data is available in open-access at [50,51].

## Abbreviations

3DVA	3-dimensional vector antenna
5G	Fifth generation of cellular communications
4G	Fourth generation of cellular communications
ANN	Artificial Neural Networks
AoA	Angle of Arrival
ASMGCS	Advanced Surface Movement Guidance and Control Systems
ATM	Air Traffic Management
AWGN	Additive White Gaussian Noise
BS	Base Station
CDL	Clustered Delay Line
CIR	Channel Impulse Response
CNN	Convolutional Neural Networks
CSIRS	Channel Status Information Reference Signal
DL	Downlink
ESPRIT	Estimation of Signal Parameters via Rotational Invariance Techniques
FFT	Fast Fourier Transform
GI	Guard Interval
GNSS	Global Navigation Satellite Systems
LOS	Line of Sight
MIMO	Multiple Input Multiple Output
MISO	Multiple Input Single Output
ML	Machine Learning
MLAT	Multilateration
MUSIC	MUltiple SIgnal Classification
MVDR	Minimum Variance Distortion-less Response
NLOS	Non Line of Sight
NN	Neural Networks
NR	New Radio
OFDM	Orthogonal Frequency Division Multiplexing
PRS	Positioning Reference Signals
QAM	Quadrature Amplitude Modulation
RF	Random Forest
RSS	Received Signal Strength
SIMO	Single Input Multiple Output
SISO	Single Input Single Output
SMR	Surface Movements Radar
SNR	Signal to Noise Ratio
SRS	Sounding Reference Signals
SVM	Support Vector Machines
ToA	Time of Arrival
TDL	Tapped Delay Line
TK	Teager-Kaiser transform
UE	User Equipment
UL	Uplink
ULA	Uniform Linear Array
UWB	Ultra Wide-Band
V2V	Vehicle-to-Vehicle
USRP	Universal Software Radio Peripheral
3DVA	3D vector antenna
